# Supplementary material for: A tunable LIC1-adaptor interaction modulates dynein activity in a cargo-specific manner
Source: Nat Commun. 2020 Nov 10;11:5695. doi: 10.1038/s41467-020-19538-7 (PMC7655957; doi:10.1038/s41467-020-19538-7)
Supplement: Supplementary file 1 — Supplementary Information [file 41467_2020_19538_MOESM1_ESM.pdf]

A tunable LIC1-adaptor interaction modulates dynein activity in a cargo-specific manner

In-Gyun Lee[1,3], Sydney E. Cason[1,2], Saif S. Alqassim[1,4], Erika L. F. Holzbaur[1,2], Roberto Dominguez[1],*

[1] Department of Physiology and Pennsylvania Muscle Institute, Perelman School of Medicine, University of Pennsylvania, Philadelphia, PA 19104, USA

[2] Neuroscience Graduate Group, Biomedical Graduate Studies, Perelman School of Medicine, University of Pennsylvania, Philadelphia, PA 19104, USA

[3] Present address: Korea Institute of Science and Technology (KIST), 5 Hwarangro 14-gil, Seongbuk-gu, Seoul 02792, Republic of Korea

[4] Present address: College of Medicine, Mohammed Bin Rashid University of Medicine and Health Sciences, Dubai, United Arab Emirates

*Correspondence should be addressed to R.D. (email:droberto@pennmedicine.upenn.edu)

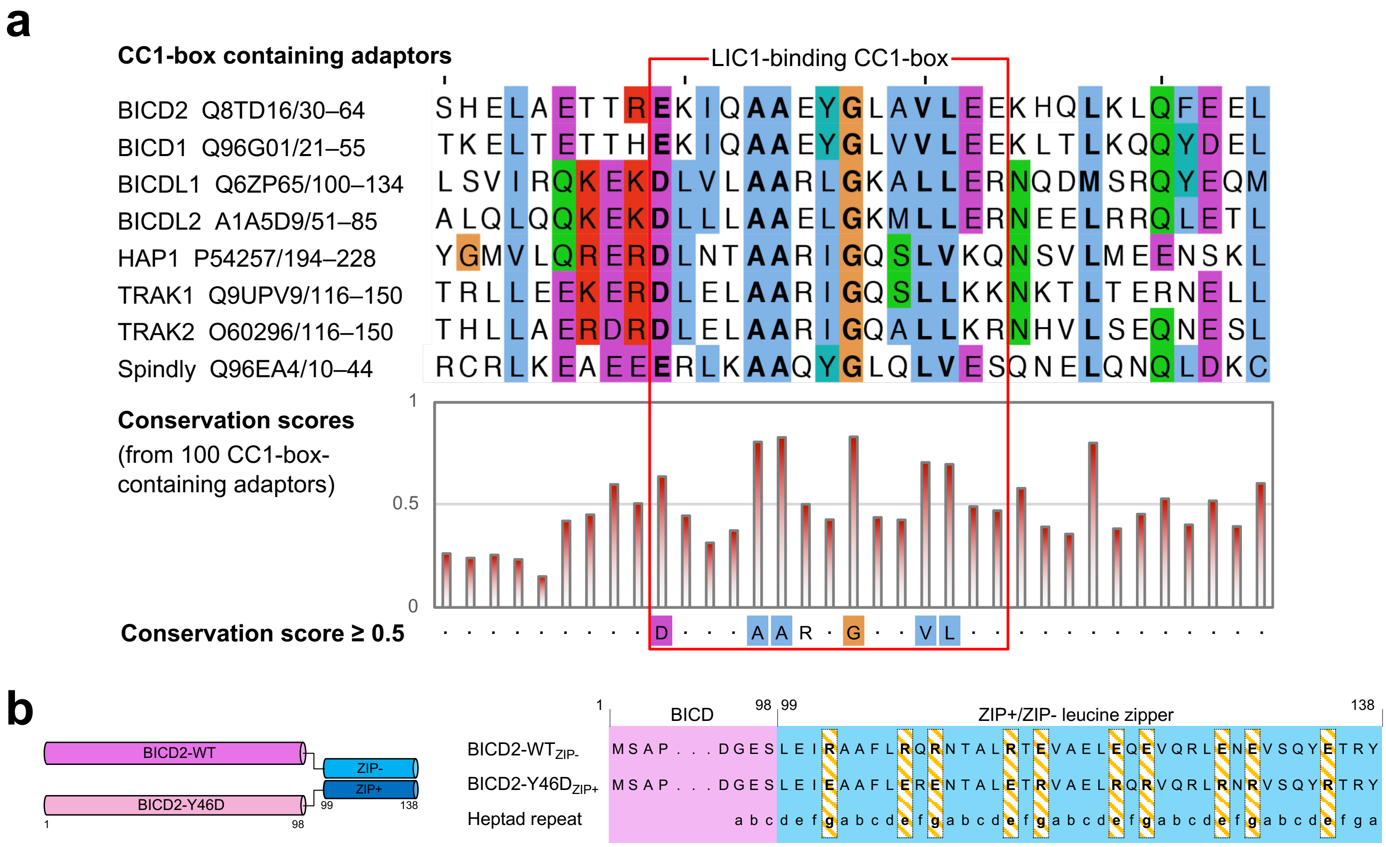

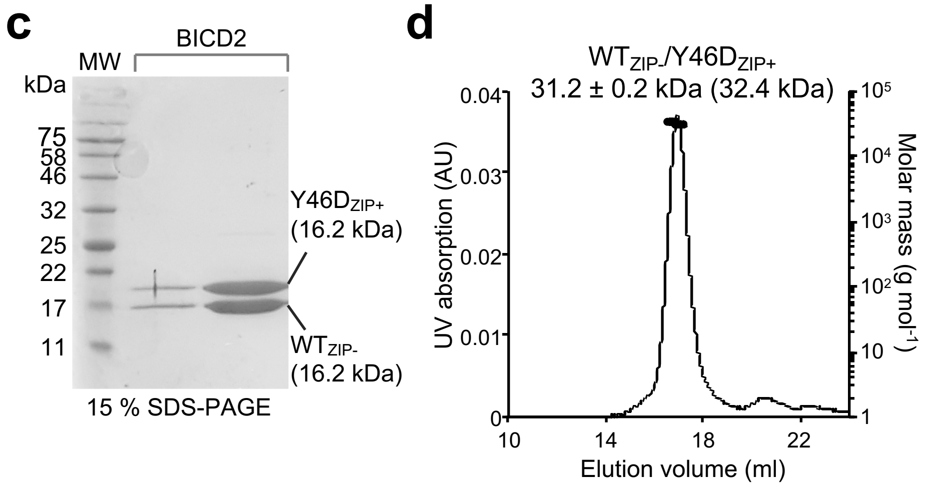

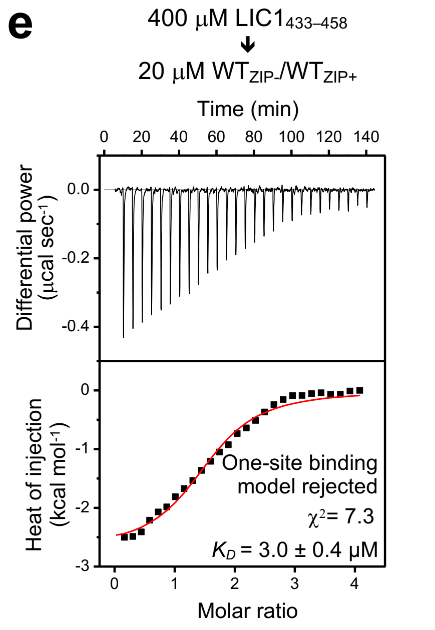

Supplementary Figure 1. The LIC1 helix binds on both side of the CC1-box coiled coil. **(a)** Sequence alignment of the CC1-box region of several human adaptors (UniProt accession codes are listed with the name of each sequence). The graph below the alignment shows the per-residue conservation scores from analysis of 100 sequences of CC1-box-containing adaptors. Amino acids with conservations scores ≥ 50% are highlighted bold. (**b**) Design of BICD2 WT/Y46D heterodimers stabilized via the C-terminal addition of the ZIP+/ZIP- leucine zipper [24]. The sequence alignment illustrates how electrostatic interactions between positively- and negatively-charged amino acids at alternating heptad positions *e* and *g* of the two coiled coil (orange striped boxes) stabilize the ZIP+/ZIP- heterodimer. (**c**) SDS-PAGE analysis of the purified BICD2 WT$_{ZIP-}$/Y46D$_{ZIP+}$ heterodimer (representative gel from n=2 independent experiments showing similar results). The two chains of the heterodimer have the same mass but migrate differently due to their different overall charges. (**d**) SEC-MALS analysis of the purified BICD2 WT$_{ZIP-}$/Y46D$_{ZIP+}$ heterodimer. The molar mass determined from light scattering (31.2 ± 0.2 kDa, right *y*-axis) and the UV absorption at 280 nm (left *y*-axis) are plotted as a function of the elution volume. (**e**) Fitting of the ITC titration of LIC1$_{433–458}$ into BICD2 WT$_{ZIP-}$/WT$_{ZIP+}$ using a one-site binding model. As indicated by the poor fit and high χ^2 value, the titration fits better to a two-site binding model with χ^2 values of 0.74 (**Fig. 1f**, left) compared to χ^2=7.3 for the one-site binding model.

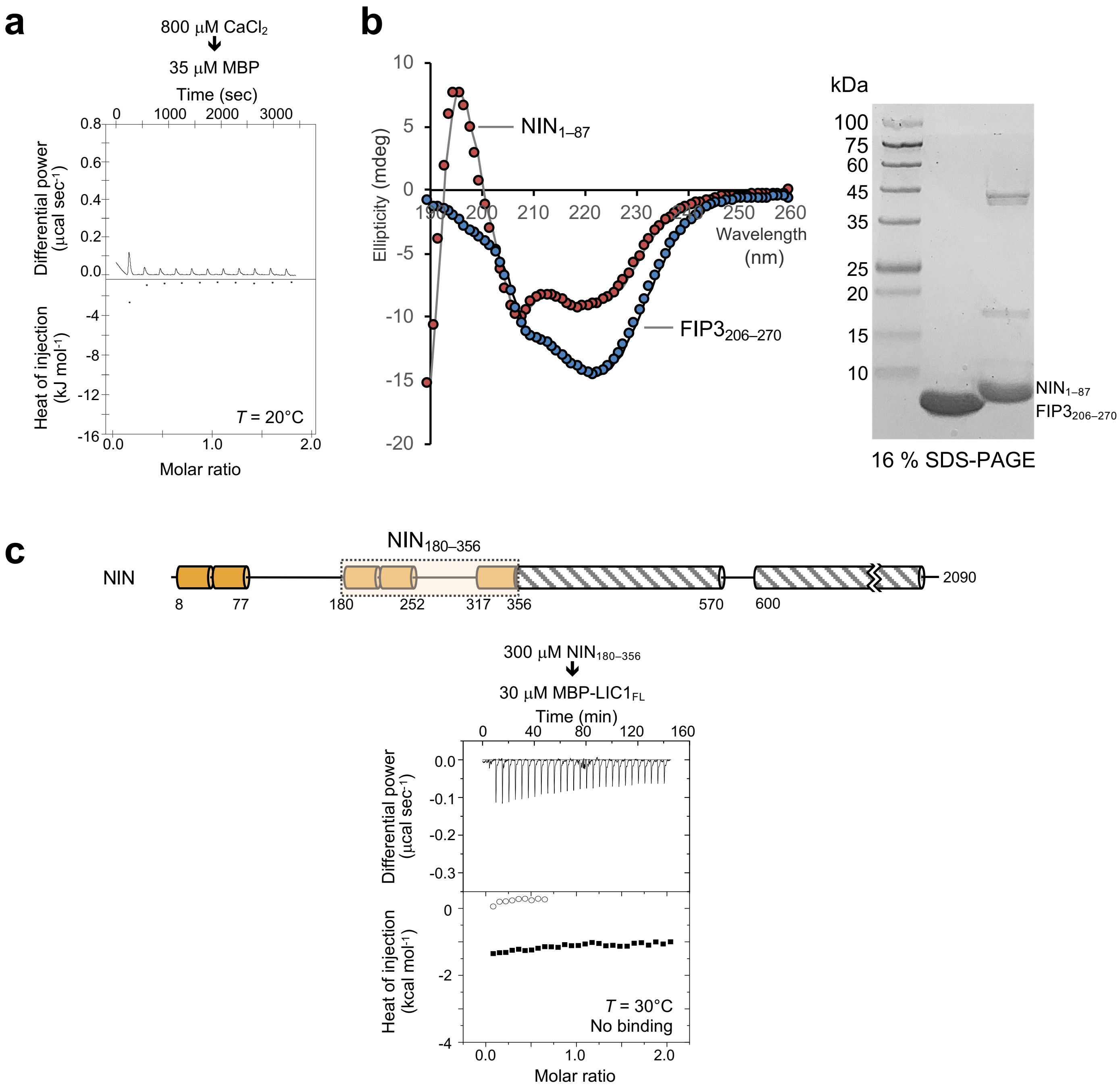

Supplementary Figure 2. Additional experiments to characterize the interaction of LIC1 with EF-hand-containing adaptors. (**a**) ITC titration of $CaCl_2$ into maltose-binding protein (MBP). The experimental conditions are listed in the figure. The lack of change in the heat of injection indicates lack of binding and precludes fitting to a binding isotherm. (**b**) Circular dichroism (CD) spectra and SDS-PAGE analysis of constructs $FIP3_{206–270}$ and $NIN_{1–87}$ (representative gel from n=2 independent experiments showing similar results). The CD spectra of both proteins are characteristic of α-helical structures, with minima around 222 and 208 nm and no evidence of unfolding. (**c**) ITC titration of $NIN_{180–356}$ (containing three EF-hands, as shown in the diagram above) into $MBP\text{-}LIC1_{FL}$. The experimental conditions are listed in the figure. Open symbols correspond to a control titration into buffer. The lack of change in the heat of injection indicates lack of binding and precludes fitting to a binding isotherm.

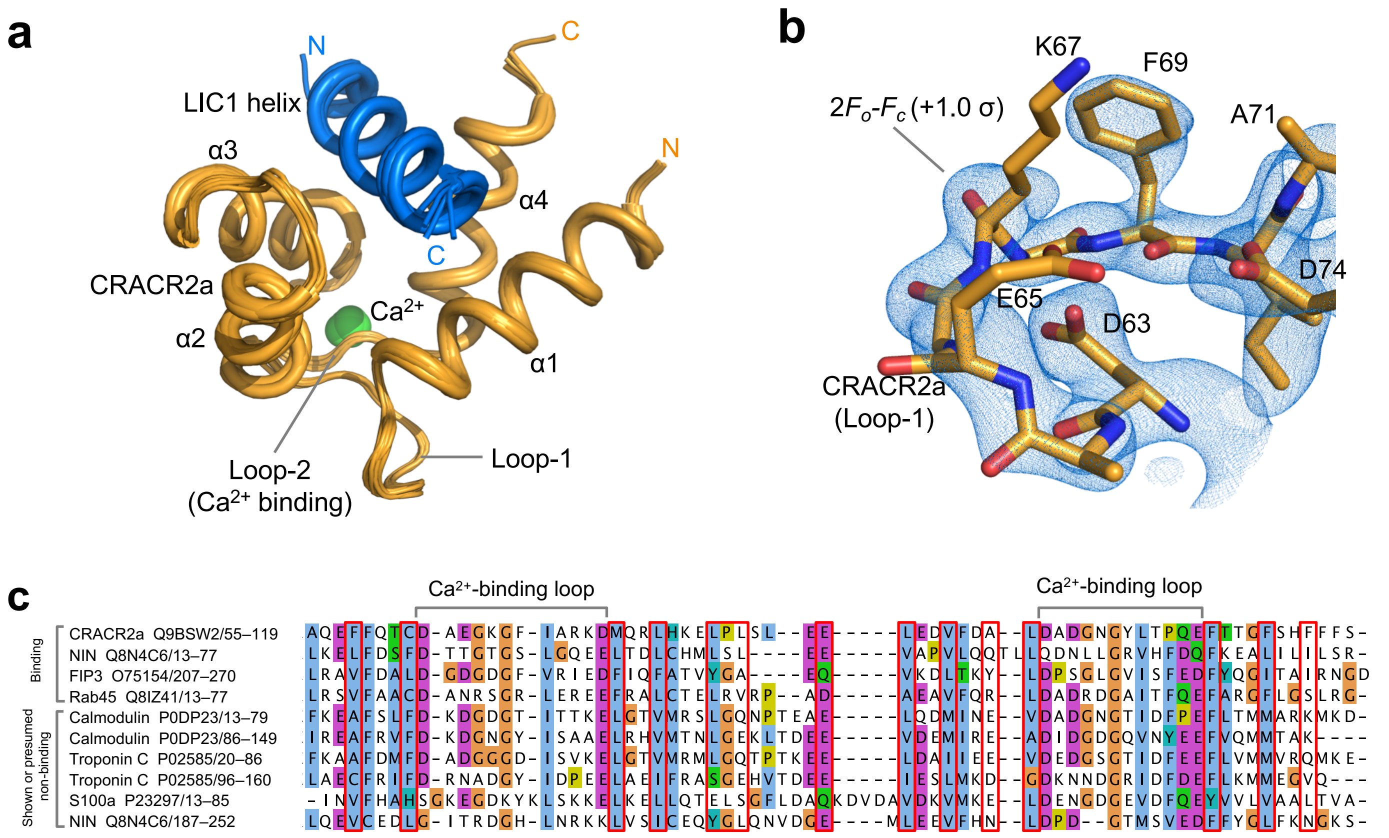

Supplementary Figure 3. Interaction of CRACR2a with LIC1. (**a**) Superimposition of the structures of the eight complex of CRACR2a$_{47–122}$ (orange) with LIC1$_{433–458}$ (blue) present in the asymmetric unit of the crystals (RMSDs < 0.4 Å for equivalent Cα atoms among the eight complexes). (**b**) Close-up view of the first EF-hand loop (Loop-1 in part a) of CRACR2a that does not bind Ca^{2+}. The 2*F*o-*F*c electron density map contoured at 1.0 σ is shown as a blue mesh. (**c**) Comparison of the sequences of EF-hand pairs that either bind (top) or are presumed not to bind (bottom) the LIC1 helix. Note that only of NIN$_{180–356}$ (containing three EF-hand domains) has been experimentally shown not to bind the LIC1 helix (**Supplementary Fig. 2c**). Amino acids boxed red are found in the vicinity of the LIC1 helix in the structure of its complex with CRACR2a. We cannot identify any pattern of conservation among these residues that would allow us to predict whether an EF-hand pair can bind the LIC1 helix. The name of each sequence includes the UniProt accession code and the range of amino acids shown in the alignment.

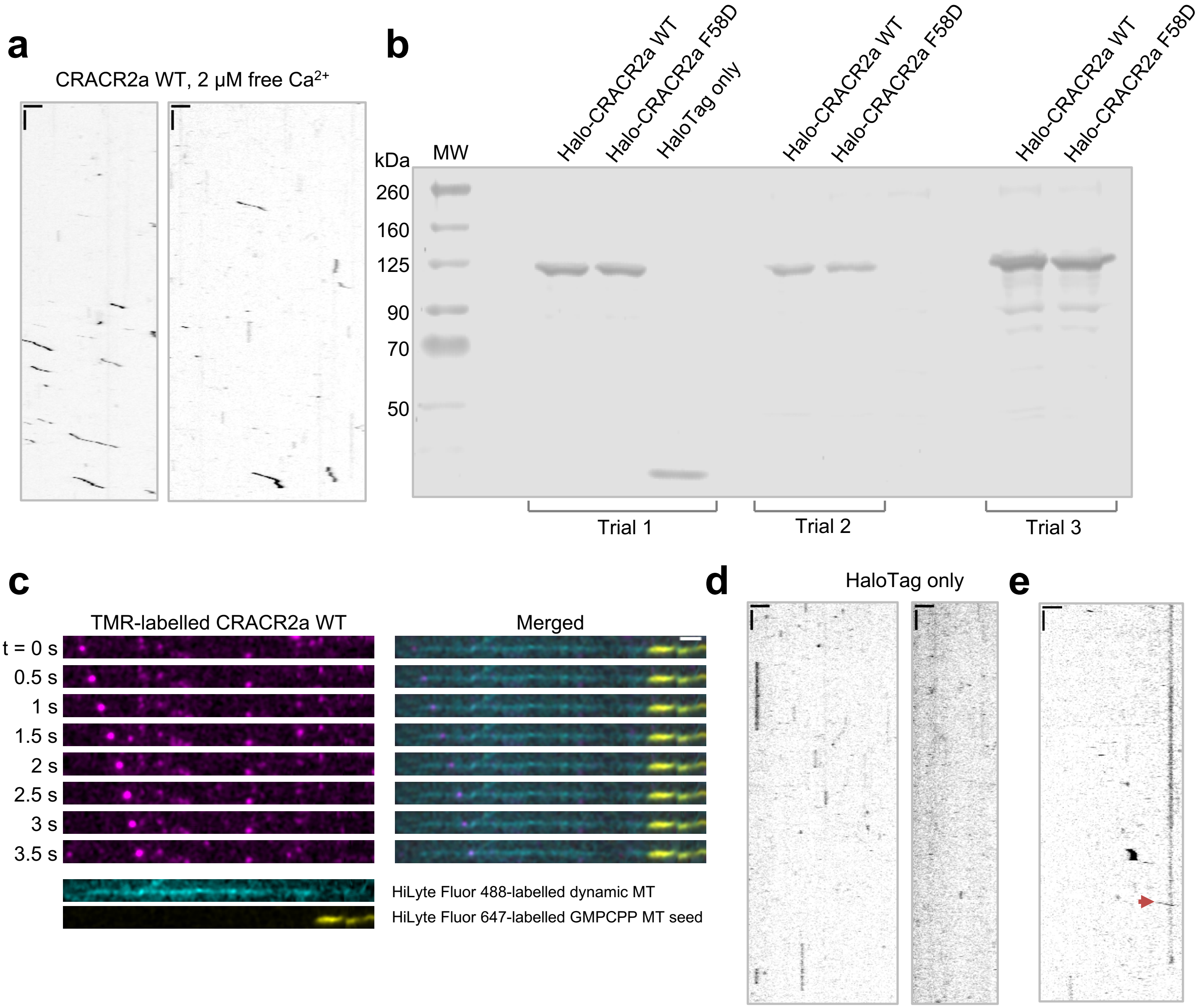

Supplementary Figure 4. CRACR2a-containing complexes move toward the microtubule minus end. (**a**) Additional representative kymographs (see also Fig. 5b in the main text) of the dynein-dynactin driven motility of single Halo-CRACR2a-positive particles (WT) analyzed by TIRF microscopy in the presence of 2 µM free Ca^{2+}. (**b**) Western blot analysis of lysates of cells expressing either WT or mutant F58D TMR-labeled Halo-CRACR2a and from n=3 biological replicates used in single molecule motility assays. The blot was probed with polyclonal anti-Halo antibody (Promega, Madison, WI) at 1:1000 dilution and analyzed using an Odyssey CLx fluorescence imaging system (LI-COR Biosciences, Lincoln, NE). Source data provided in the Source Data file. (**c**) Time-lapse images of a TMR-labeled WT CRACR2a particle (magenta) moving along a HiLyte Fluor 488-labeled dynamic microtubule (MT; cyan) toward the HiLyte Fluor 647-labelled GMPCPP MT seed (yellow) at the minus end. The bright CRACR2a particle can be seen moving towards the MT minus end in both the merged (right) or TMR (561 nm, left) channels. Scale bar, 3 µm. (**d**) Kymographs from a chamber containing TMR-labelled HaloTag alone, illustrating the lack of directed movement in the absence of CRACR2a (negative control). (**e**) Kymograph showing a single motile event (red arrow) with F58D mutant CRACR2a in the presence of Ca^{2+}. Scale bars: horizontal, 3 µm; vertical, 5 sec.

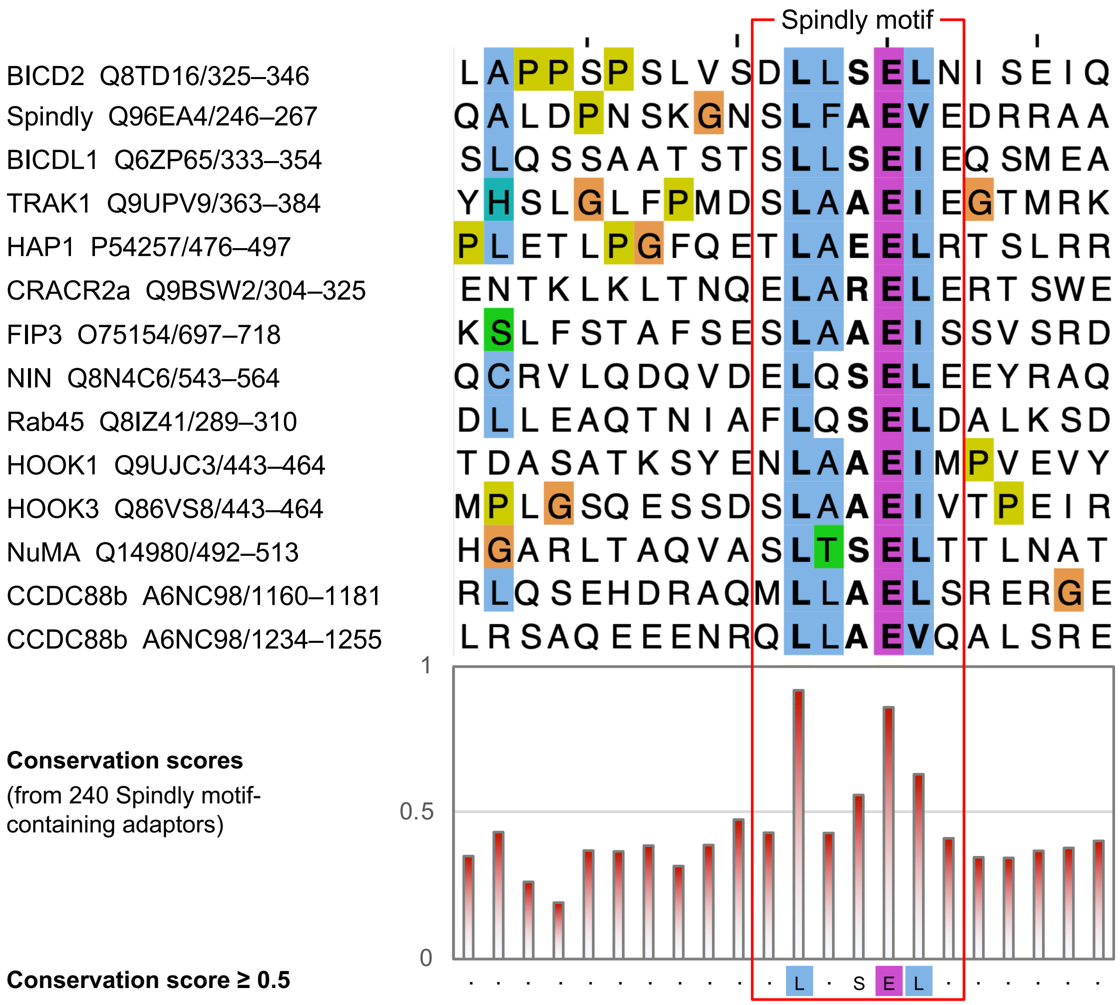

Supplementary Figure 5. The Spindly motif. Sequence alignment of the Spindly motif of several human adaptors. The UniProt accession codes and the range of amino acids shown in the alignment are listed with the name of each sequence. The graph below the alignment shows the per-residue conservation scores from analysis of 240 sequences of Spindly motif-containing adaptors. Amino acids with conservation scores ≥ 50% are highlighted (bold).

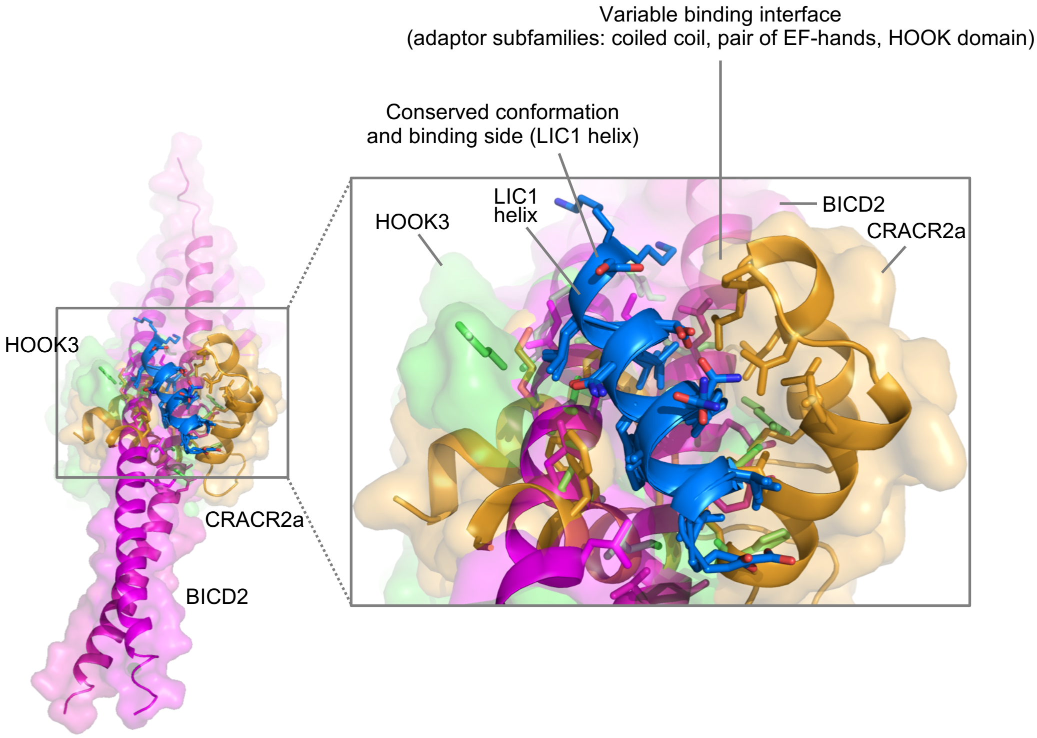

Supplementary Figure 6. The LIC1-adaptor interface is conserved on the LIC1 side but variable on the adaptor side. Superimposition of the three structures of adaptor-LIC1 complexes, including overall view (left) and close-up view (right) of the LIC1 helix (blue) binding interface. HOOK3, BICD2 and CRACR2a are colored green, magenta and orange, respectively. While the conformation and orientation of side chains is remarkably well-conserved for the LIC1 helix, there is no specific conservation of the binding interface among different adaptors subfamilies, other than the overall hydrophobic character of the interface.

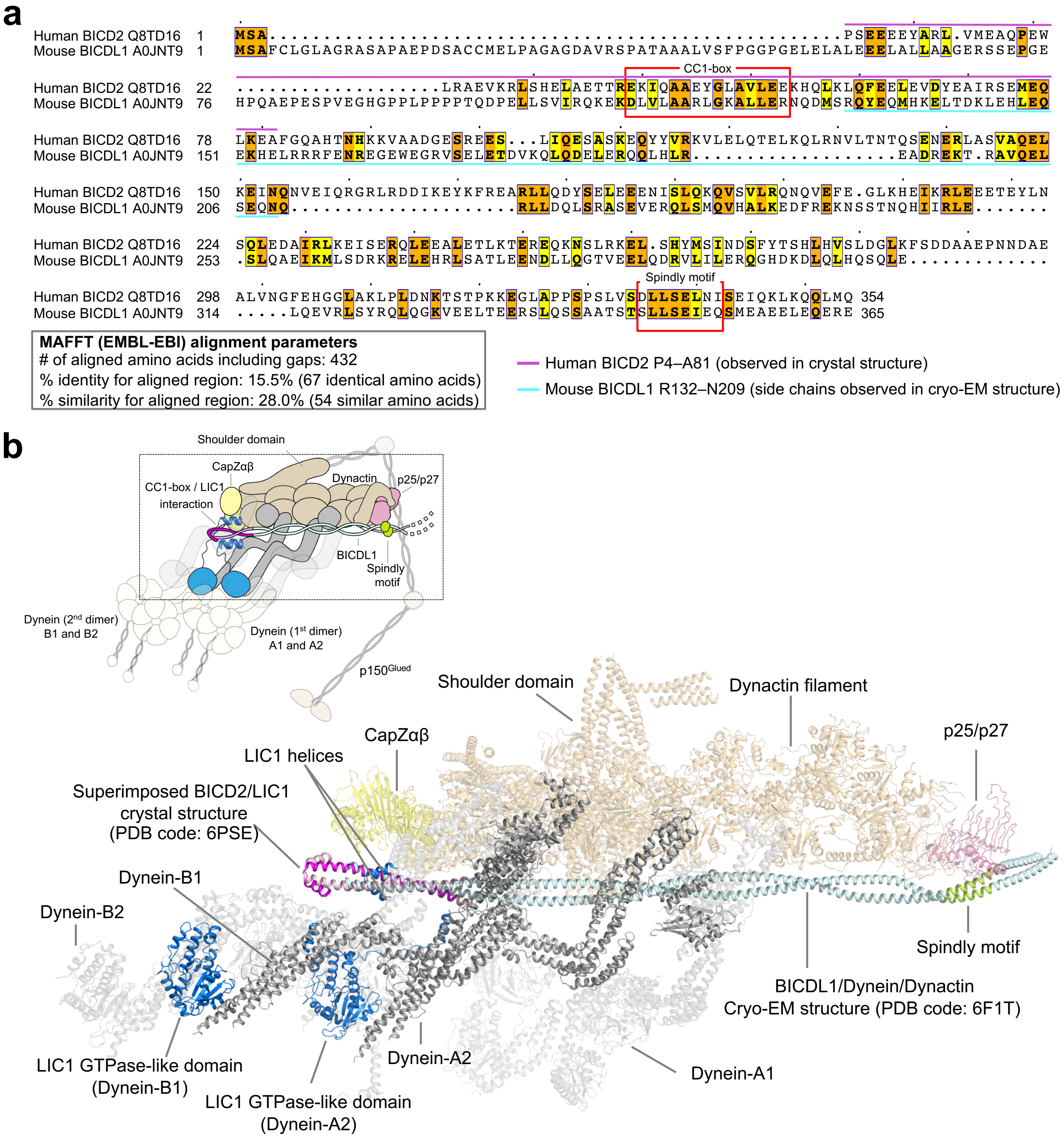

Supplementary Figure 7. Interaction of the LIC1 helix with CC1-box-containing adaptors within the adaptor-dynein-dynactin complex. (**a**) Sequence alignment of the dynactin-binding region of human BICD2 and mouse BICDL1. The sequence identity is low (16%), but identical residues (bold) are identified along the length of the alignment, including within the CC1-box and Spindly motif (red boxes). A magenta line indicates the BICD2 region observed in the crystal structure of BICD2$_{1\text{-}98}$-LIC1$_{433\text{-}458}$. A cyan line below the alignment indicates the BICDL1 region for which side chains were observed in the 3.5-Å cryo-EM structure of BICDL1-dynein-dynactin [9] (residues 132–209; PDB code: 6PSE). (**b**) Cartoon (top) and atomic model (bottom) representations of the crystal structure of BICD2$_{1\text{-}98}$-LIC1$_{433\text{-}458}$ superimposed onto the cryo-EM structure of BICDL1-dynein-dynactin based on the alignment shown in part a. Relevant subunits and interactions are labeled. The cryo-EM structure contains two dynein dimers bound (A and B). Based on the positions of the four LIC1 chains, it appears likely that each dynein dimer contributes one LIC1 helix (blue) to the interaction with the CC1-box, possibly explaining how two dynein dimers can be simultaneously activated by a single adaptor coiled coil dimer.

Supplementary Table 1. Dynein-dynactin adaptors and their cargoes

Subfamily	Adaptor	Evidence of LIC1 binding	Evidence of dynein-dynactin binding/activation	Confirmed or unknown activator	Cellular cargo
CC1-box-containing adaptors					
BICD family	BICD1	UNK	Co-IP[1]	UNK	Golgi-derived vesicles[2]
	BICD2	Co-IP[3,4,5] ITC[6], SPR[5]	TIRF (Reconstituted)[7,8,9,10] Relocation assay[11], TIRF (Lysate)[12]	Confirmed	Golgi-derived vesicles[2,13], Nuclear pore complexes[14], Viral particles[15]
	BICDL1 (BICDR1)	UNK	TIRF (Reconstituted)[10,16], Relocation assay[11]	Confirmed	Rab6 vesicles[17]
	BICDL2 (BICDR1)	UNK	Homology to BICDL1	UNK	Rab13 vesicles[17]
	Spindly	Co-IP[3,5], SPR[5]	TIRF (Co-IP)[8]	Confirmed	Kinetochore[3]
TRAK family	TRAK1	Co-IP[5]	Co-IP[18]	UNK	Mitochondria[18]
	TRAK2	UNK	Co-IP[18]	UNK	Mitochondria[18]
	HAP1	UNK	Co-IP[19,20,21]	UNK	Autophagosomes[22], BDNF-containing vesicles[23]
HOOK domain-containing adaptors					
HOOK family	HOOK1	ITC[6]	TIRF (Lysate)[12]	Confirmed	Early endosomes[24,25], Clathrin-independent cargoes[26]
	HOOK2	Co-IP[27]	Relocation assay[27]	UNK	Early endosomes[25], Spermatid intramanchette[28], Centrosomal proteins[29]
	HOOK3	Co-IP[5,30], ITC[6], SPR[5]	TIRF (Reconstituted)[8,9,16] TIRF (Lysate)[6,12]	Confirmed	Early endosomes[25]
CCDC family	CCDC88A (Girdin)	UNK	Co-IP[31]	UNK	UNK
	CCDC88B (HKRP3)	UNK	Co-IP[32]	UNK	Secretory lysosomes[32]
	CCDC88C (Daple)	UNK	Co-IP[31]	UNK	Planar cell polarity proteins[33]
	NuMA	Co-IP[34]	Co-IP[32]	UNK	Spindle microtubules[35]
EF-hand-containing adaptors					
NIN family	NIN (Ninein)	Co-IP[5]	TIRF (Reconstituted)[31]	Confirmed	Centrosome[36]
	NINL (Ninein-like)	UNK	TIRF (Reconstituted)[9, 31]	Confirmed	Rab8- and MICAL3-containing vesicles[37]
	CRACR2a	UNK	TIRF (Reconstituted)[38]	Confirmed	ORAI1-STIM1 complex[39]
	Rab45	UNK	TIRF (Reconstituted)[38]	Confirmed	UNK
FIP family (Class II)	Rab11-FIP3 (FIP3)	Co-IP[4,5,40,41]	TIRF (Co-IP)[8]	Confirmed	Recycling endosomes[41]
	Rab11-FIP4 (FIP4)	UNK	UNK	UNK	Recycling endosomes[41]
Other dynein-dynactin adaptors (RH1 domain-containing)					
JIP family	JIP1	UNK	Co-IP[42]	UNK (Unlikely)	Autophagosomes[43] Components of JNK signaling pathway[44]
	JIP2	UNK	UNK	UNK (Unlikely)	Components of JNK signaling pathway[44]
	JIP3 (Sunday Driver)	UNK	Co-IP[45]	UNK (Unlikely)	Components of JNK signaling pathway[44]
	JIP4	UNK	Co-IP[46]	UNK (Unlikely)	Components of JNK signaling pathway[44]
	RILP	Co-IP[4,47]	Co-IP[48]	UNK (Unlikely)	Autophagosomes[49] Lysosomes[48]

Abbreviations: UNK, Unknown; Co-IP, Co-immunoprecipitation; ITC, Isothermal Titration Calorimetry; SPR, Surface Plasmon Resonance; BDNF, Brain-Derived Neurotrophic Factor; TIRF, Total Internal Reflection Fluorescence microscopy; MICAL3, [F-actin]-monooxygenase MICAL3; ORAI1, Ca^{2+}-Release-Activated Ca^{2+} Channel Protein-1; STIM1, Stromal Interaction Molecule-1; JNK, c-Jun N-terminal Kinase

Supplementary Table 2. Primers used in this study

Construct	Forward primer	Reverse primer
BICD2$_{1-98}$	5' cgcggatccgaaaacctgtattttcaggga atgtccgcgccgtcgga	5' ctcgtcgacttagctctcaccatcagcagcca
CRACR2a$_{47-122}$	5' cgcggatccgaaaacctgtattttcag ggaggccagctagtcatgctg	5' gcgtcgacttaattctggctgaagaagaagtgac
FIP3$_{206-270}$	5' ggaattcgagaacctgtattttcaaagccgc ctccgagccgtgttc	5' ctcgtcgacttaatctccgtttctgatggctgtg
NIN$_{180-356}$	5'cgcggatccgaaaacctgtattttcagggacaagac tggatagaagagaaactgc	5' gcgtcgacgctgttcttggtaaccaaaagttcat
NIN$_{1-87}$	5' cgcggatccgaaaacctgtattttcaggga atggatgag gtggagcagg	5' gcgtcgacttcttgaaagtgttcttcatttgac
BICD2$_{1-98}$Y46D	5' : ccgccagtccatcctcggccgcctg	5' : caggcggccgaggatggactggcgg
BICD2$_{1-98}$D46Y	5' : caggcggccgagtatggactggcgg	5' : ccgccagtccatactcggccgcctg
BICD2$_{1-98}$WT$_{ZIP-}$	5' cgggatccgaaaacctgtattttcag	Rev1: 5' gcggtgttgcgctggcgcagaaacgccgcgcgaatttccaggct ctcaccatcagcagcc
		Rev2: 5' caggcgctgcacttcctgttccagttccgccacttcggtgcgcagc gcggtgttgcgctg
		Rev3: 5' gcgtcgacttaatagcgggtttcatactggctcacttcgttttc caggcgctgcacttc
BICD2$_{1-98}$Y46D$_{ZIP+}$	5' ggattccatatggaaaacctgtattttcag	Rev1: 5' gcggtgttttcgcgttccagaaacgccgcttcaatttccag gctctcaccatcagcagcc
		Rev2: 5' gcgctgcacgcgctggcgcagttccgccacgcgggtttccagc gcggtgttttcgcgttc
		Rev3: 5' gcgtcgacttaatagcgggtgcgatactggctcacgcggttgcgc aggcgctgcacgcg
LIC1$_{FL}$	5' cgcggatccggaaaatctgtatttccaatc catggcggccgtgggg	5' acggtcgactcaagaagcttctccttccgtaggagat
LIC1$_{1-461}$	5' cgcggatccggaaaatctgtatttccaatc catggcggccgtgggg	5' ctcgtcgacgcctcctggagagccagtcttttta
CRACR2a$_{FL}$ (TIRF)	5' tagaattcagagctatggctgcccctgacgggagggta	5' tatgagctcccaggaagtccgctccag
CRACR2a$_{FL}$F58D (TIRF)	5' gaaggcacaggaggactttcagacctgtg	5' cacaggtctgaaagtcctcctgtgccttc

Supplementary References

1. Fumoto K, Hoogenraad CC, Kikuchi A. GSK-3β-regulated interaction of BICD with dynein is involved in microtubule anchorage at centrosome. *EMBO J* **25**, 5670-5682 (2006).

2. Matanis T*, et al.* Bicaudal-D regulates COPI-independent Golgi–ER transport by recruiting the dynein-dynactin motor complex. *Nat Cell Biol* **4**, 986-992 (2002).

3. Gama JB*, et al.* Molecular mechanism of dynein recruitment to kinetochores by the Rod-Zw10-Zwilch complex and Spindly. *J Cell Biol* **216**, 943-960 (2017).

4. Schroeder CM, Ostrem JM, Hertz NT, Vale RD. A Ras-like domain in the light intermediate chain bridges the dynein motor to a cargo-binding region. *eLife* **3**, e03351 (2014).

5. Celestino R*, et al.* A transient helix in the disordered region of dynein light intermediate chain links the motor to structurally diverse adaptors for cargo transport. *PLoS Biol* **17**, e3000100 (2019).

6. Lee IG, Olenick MA, Boczkowska M, Franzini-Armstrong C, Holzbaur ELF, Dominguez R. A conserved interaction of the dynein light intermediate chain with dynein-dynactin effectors necessary for processivity. *Nat Commun* **9**, 986 (2018).

7. Schlager MA, Hoang HT, Urnavicius L, Bullock SL, Carter AP. *In vitro* reconstitution of a highly processive recombinant human dynein complex. *EMBO J* **33**, 1855-1868 (2014).

8. McKenney RJ, Huynh W, Tanenbaum ME, Bhabha G, Vale RD. Activation of cytoplasmic dynein motility by dynactin-cargo adapter complexes. *Science* **345**, 337-341 (2014).

9. Htet ZM, Gillies JP, Baker RW, Leschziner AE, DeSantis ME, Reck-Peterson SL. Lis1 promotes the formation of activated cytoplasmic dynein-1 complexes. *Nat Cell Biol* **22**, 518–525 (2020).

10. Elshenawy MM*, et al.* Cargo adaptors regulate stepping and force generation of mammalian dynein-dynactin. *Nat Chem Biol* **15**, 1093-1101 (2019).

11. Schlager MA*, et al.* Bicaudal D family adaptor proteins control the velocity of dynein-based movements. *Cell Rep* **8**, 1248-1256 (2014).

12. Olenick MA, Tokito M, Boczkowska M, Dominguez R, Holzbaur EL. Hook adaptors induce unidirectional processive motility by enhancing the dynein-dynactin interaction. *J Biol Chem* **291**, 18239-18251 (2016).

13. Belyy V, Schlager MA, Foster H, Reimer AE, Carter AP, Yildiz A. The mammalian dynein-dynactin complex is a strong opponent to kinesin in a tug-of-war competition. *Nat Cell Biol* **18**, 1018 (2016).

14. Splinter D*, et al.* Bicaudal D2, dynein, and kinesin-1 associate with nuclear pore complexes and regulate centrosome and nuclear positioning during mitotic entry. *PLoS Biol* **8**, e1000350 (2010).

15. Dharan A*, et al.* Bicaudal D2 facilitates the cytoplasmic trafficking and nuclear import of HIV-1 genomes during infection. *Proc Natl Acad Sci USA* **114**, E10707-E10716 (2017).

16. Urnavicius L*, et al.* Cryo-EM shows how dynactin recruits two dyneins for faster movement. *Nature* **554**, 202 (2018).

17. Schlager MA*, et al.* Pericentrosomal targeting of Rab6 secretory vesicles by Bicaudal-D-related protein 1 (BICDR-1) regulates neuritogenesis. *EMBO J* **29**, 1637-1651 (2010).

18. van Spronsen M*, et al.* TRAK/Milton motor-adaptor proteins steer mitochondrial trafficking to axons and dendrites. *Neuron* **77**, 485-502 (2013).

19. Engelender S*, et al.* Huntingtin-associated protein 1 (HAP1) Interacts with the p150^{Glued} subunit of dynactin. *Hum Mol Genet* **6**, 2205-2212 (1997).

20. Li S-H, Gutekunst C-A, Hersch SM, Li X-J. Interaction of huntingtin-associated protein with dynactin P150^{Glued}. *J Neurosci* **18**, 1261-1269 (1998).

21. Caviston JP, Ross JL, Antony SM, Tokito M, Holzbaur EL. Huntingtin facilitates dynein/dynactin-mediated vesicle transport. *Proc Natl Acad Sci USA* **104**, 10045-10050 (2007).

22. Wong YC, Holzbaur EL. The regulation of autophagosome dynamics by huntingtin and HAP1 is disrupted by expression of mutant huntingtin, leading to defective cargo degradation. *J Neurosci* **34**, 1293-1305 (2014).

23. Lim Y*, et al.* HAP1 is required for endocytosis and signalling of BDNF and its receptors in neurons. *Mol Neurobiol* **55**, 1815-1830 (2018).

24. Guo X, Farías GG, Mattera R, Bonifacino JS. Rab5 and its effector FHF contribute to neuronal polarity through dynein-dependent retrieval of somatodendritic proteins from the axon. *Proc Natl Acad Sci USA* **113**, E5318-E5327 (2016).

25. Xu L, Sowa ME, Chen J, Li X, Gygi SP, Harper JW. An FTS/Hook/p107FHIP complex interacts with and promotes endosomal clustering by the homotypic vacuolar protein sorting complex. *Mol Biol Cell* **19**, 5059-5071 (2008).

26. Maldonado-Baez L, Cole NB, Kramer H, Donaldson JG. Microtubule-dependent endosomal sorting of clathrin-independent cargo by Hook1. *J Cell Biol* **201**, 233-247 (2013).

27. Dwivedi D, Kumari A, Rathi S, Mylavarapu SV, Sharma M. The dynein adaptor Hook2 plays essential roles in mitotic progression and cytokinesis. *J Cell Biol* **218**, 871-894 (2019).

28. Okuda H, DeBoer K, O'Connor AE, Merriner DJ, Jamsai D, O'Bryan MK. LRGUK1 is part of a multiprotein complex required for manchette function and male fertility. *FASEB J* **31**, 1141-1152 (2017).

29. Szebenyi G, Hall B, Yu R, Hashim AI, Kramer H. Hook2 localizes to the centrosome, binds directly to centriolin/CEP110 and contributes to centrosomal function. *Traffic* **8**, 32-46 (2007).

30. Schroeder CM, Vale RD. Assembly and activation of dynein–dynactin by the cargo adaptor protein Hook3. *J Cell Biol* **214**, 309-318 (2016).

31. Redwine WB*, et al.* The human cytoplasmic dynein interactome reveals novel activators of motility. *eLife* **6**, e28257 (2017).

32. Ham H, Huynh W, Schoon RA, Vale RD, Billadeau DD. HkRP3 is a microtubule-binding protein regulating lytic granule clustering and NK cell killing. *J Immunol* **194**, 3984-3996 (2015).

33. Siletti K, Tarchini B, Hudspeth A. Daple coordinates organ-wide and cell-intrinsic polarity to pattern inner-ear hair bundles. *Proc Natl Acad Sci USA* **114**, E11170-E11179 (2017).

34. Renna C*, et al.* Organizational principles of the NuMA-dynein interaction interface and implications for mitotic spindle functions. *Structure*, in press (2020).

35. Hueschen CL, Kenny SJ, Xu K, Dumont S. NuMA recruits dynein activity to microtubule minus-ends at mitosis. *eLife* **6**, e29328 (2017).

36. Mogensen MM, Malik A, Piel M, Bouckson-Castaing V, Bornens M. Microtubule minus-end anchorage at centrosomal and non-centrosomal sites: the role of Ninein. *J Cell Sci* **113**, 3013-3023 (2000).

37. Bachmann-Gagescu R*, et al.* The ciliopathy protein CC2D2A associates with NINL and functions in RAB8-MICAL3-regulated vesicle trafficking. *PLoS Genet* **11**, (2015).

38. Wang Y, Huynh W, Skokan TD, Lu W, Weiss A, Vale RD. CRACR2a is a calcium-activated dynein adaptor protein that regulates endocytic traffic. *J Cell Biol*, (2019).

39. Srikanth S, Jung H-J, Kim K-D, Souda P, Whitelegge J, Gwack Y. A novel EF-hand protein, CRACR2A, is a cytosolic Ca^{2+} sensor that stabilizes CRAC channels in T cells. *Nat Cell Biol* **12**, 436-446 (2010).

40. Johansson M*, et al.* Activation of endosomal dynein motors by stepwise assembly of Rab7-RILP-$p150^{Glued}$, ORP1L, and the receptor βIII spectrin. *J Cell Biol* **176**, 459-471 (2007).

41. Horgan CP, Hanscom SR, Jolly RS, Futter CE, McCaffrey MW. Rab11-FIP3 links the Rab11 GTPase and cytoplasmic dynein to mediate transport to the endosomal-recycling compartment. *J Cell Sci* **123**, 181-191 (2010).

42. Fu M-m, Holzbaur EL. JIP1 regulates the directionality of APP axonal transport by coordinating kinesin and dynein motors. *J Cell Biol* **202**, 495-508 (2013).

43. Fu M-m, Nirschl JJ, Holzbaur EL. LC3 binding to the scaffolding protein JIP1 regulates processive dynein-driven transport of autophagosomes. *Dev Cell* **29**, 577-590 (2014).

44. Dhanasekaran D, Kashef K, Lee C, Xu H, Reddy E. Scaffold proteins of MAP-kinase modules. *Oncogene* **26**, 3185-3202 (2007).

45. Cavalli V, Kujala P, Klumperman J, Goldstein LS. Sunday driver links axonal transport to damage signaling. *J Cell Biol* **168**, 775-787 (2005).

46. Montagnac G*, et al.* ARF6 Interacts with JIP4 to control a motor switch mechanism regulating endosome traffic in cytokinesis. *Curr Biol* **19**, 184-195 (2009).

47. Scherer J, Yi J, Vallee RB. PKA-dependent dynein switching from lysosomes to adenovirus: a novel form of host-virus competition. *J Cell Biol* **205**, 163-177 (2014).

48. Jordens I*, et al.* The Rab7 effector protein RILP controls lysosomal transport by inducing the recruitment of dynein-dynactin motors. *Curr Biol* **11**, 1680-1685 (2001).

49. Khobrekar NV, Quintremil S, Dantas TJ, Vallee RB. The Dynein adaptor RILP controls neuronal autophagosome biogenesis, transport, and clearance. *Dev Cell* **53**, 141-153 (2020).
