# Supplementary material for: A tunable LIC1-adaptor interaction modulates dynein activity in a cargo-specific manner
Source: Nat Commun. 2020 Nov 10;11:5695. doi: 10.1038/s41467-020-19538-7 (PMC7655957; doi:10.1038/s41467-020-19538-7)
Supplement: Supplementary file 3 — Reporting Summary [file 41467_2020_19538_MOESM3_ESM.pdf]

nature research

Corresponding author(s): Roberto Dominguez

Last updated by author(s): Oct 5, 2020

Reporting Summary

Nature Research wishes to improve the reproducibility of the work that we publish. This form provides structure for consistency and transparency in reporting. For further information on Nature Research policies, see our Editorial Policies and the Editorial Policy Checklist.

Statistics

For all statistical analyses, confirm that the following items are present in the figure legend, table legend, main text, or Methods section.

n/a	Confirmed	
☐	☒	The exact sample size (n) for each experimental group/condition, given as a discrete number and unit of measurement
☐	☒	A statement on whether measurements were taken from distinct samples or whether the same sample was measured repeatedly
☐	☒	The statistical test(s) used AND whether they are one- or two-sided<br>*Only common tests should be described solely by name; describe more complex techniques in the Methods section.*
☒	☐	A description of all covariates tested
☒	☐	A description of any assumptions or corrections, such as tests of normality and adjustment for multiple comparisons
☐	☒	A full description of the statistical parameters including central tendency (e.g. means) or other basic estimates (e.g. regression coefficient) AND variation (e.g. standard deviation) or associated estimates of uncertainty (e.g. confidence intervals)
☐	☒	For null hypothesis testing, the test statistic (e.g. F, t, r) with confidence intervals, effect sizes, degrees of freedom and P value noted<br>*Give P values as exact values whenever suitable.*
☒	☐	For Bayesian analysis, information on the choice of priors and Markov chain Monte Carlo settings
☒	☐	For hierarchical and complex designs, identification of the appropriate level for tests and full reporting of outcomes
☒	☐	Estimates of effect sizes (e.g. Cohen's d, Pearson's r), indicating how they were calculated

Our web collection on statistics for biologists contains articles on many of the points above.

Software and code

Policy information about availability of computer code

Data collection	X-ray diffraction data for BICD2-LIC1 complex were collected at the Stanford Synchrotron Radiation Lightsource (SSRL) beamline 14-1.<br>X-ray diffraction data for CRACR2a-LIC1 complex were colelcted at the Macromolecular x-ray science at the Cornell High Energy Synchrotron Source (MacCHESS) beamline F1. Functional assays were collected using commercial software Volocity (PerkinElmer; v. 6)
Data analysis	1. The crystallographic diffraction datasets were indexed, integrated, and scaled using software HKL2000 (v. 0.98.691g).<br>2. The structures were determined using the single-wavelength anomalous dispersion method with the program SnB (v. 2.3)<br>3. Model building was carried out with the program Coot (v.0.8.2) .The structures of BICD2-LIC1 and CRACR2a-LIC1 were refined with the programs Phenix (v. 1.16.3549) and Refmac5 (v. 5.8.0131), respectively.<br>4. Structure figures were generated with the program PyMOL (v.1.6.0.0).<br>5. Sequence alignments were generated with the program MAFFT (v.6.240), and illustrated using either Jalview (v.2.10.3b1) or ESPript (v.3.0). The conservation scores of amino acids in sequence alignments were determined with the program Scorecons server (v.2002).<br>6. Multiangle-scattering analysis data were analyzed using the ASTRA software (v.5.3.1).<br>7. Functional assays were analyzed using ImageJ (v. 2.1.0/1.53c), commercial software Prism 6.0-8.0 (GraphPad), and Microsoft Excel for Mac (v. 16.41).

For manuscripts utilizing custom algorithms or software that are central to the research but not yet described in published literature, software must be made available to editors and reviewers. We strongly encourage code deposition in a community repository (e.g. GitHub). See the Nature Research guidelines for submitting code & software for further information.

Data

Policy information about availability of data

All manuscripts must include a data availability statement. This statement should provide the following information, where applicable:

- Accession codes, unique identifiers, or web links for publicly available datasets
- A list of figures that have associated raw data
- A description of any restrictions on data availability

Atomic coordinates and structure factor amplitudes for the structures of BICD2-LIC1 and CRACR2a-LIC1 were deposited with the Protein Data Bank (PDB) under accession codes 6PSE and 6PSD, respectively. The Other data and materials are available from the corresponding author upon reasonable request.

Field-specific reporting

Please select the one below that is the best fit for your research. If you are not sure, read the appropriate sections before making your selection.

☒ Life sciences ☐ Behavioural & social sciences ☐ Ecological, evolutionary & environmental sciences

For a reference copy of the document with all sections, see nature.com/documents/nr-reporting-summary-flat.pdf

Life sciences study design

All studies must disclose on these points even when the disclosure is negative.

Sample size	The sample size (n) of each experiment is provided in the corresponding figure captions in the main manuscript and supplementary information. Sample sizes are sufficient given the significant differences and large effect sizes seen between conditions.
Data exclusions	Crystallographic diffraction images with weak, unreliable reflections were excluded from the dataset. Exclusion criteria was not pre-estabiished. Otherwise, no data were excluded from analysis.
Replication	All ITC experiments presented in this study were repeated at least two times. Functional assays were repeated five times. All attempts at replication were successful.
Randomization	No randomization was required because the results of biochemical measurements, structural determination, and motility assays are not affected by sample randomization.
Blinding	Blinding is not relevant for structure determination since the result are not subjective. Investigators were not blinded during functional assays because the results are affected by the knowledge of sample identities.

Reporting for specific materials, systems and methods

We require information from authors about some types of materials, experimental systems and methods used in many studies. Here, indicate whether each material, system or method listed is relevant to your study. If you are not sure if a list item applies to your research, read the appropriate section before selecting a response.

Materials & experimental systems

n/a	Involved in the study	
☐	☒	Antibodies
☐	☒	Eukaryotic cell lines
☒	☐	Palaeontology and archaeology
☒	☐	Animals and other organisms
☒	☐	Human research participants
☒	☐	Clinical data
☒	☐	Dual use research of concern

Methods

n/a	Involved in the study	
☒	☐	ChIP-seq
☒	☐	Flow cytometry
☒	☐	MRI-based neuroimaging

Antibodies

Antibodies used	Anti-Halo-antibody (Promega, G9281)
Validation	Anti-Halo-antibody (Promega, G9281) is a purified rabbit polyclonal antibody raised against the HaloTag® protein. The antibody is purified using Protein G affinity resin and supplied at 1mg/ml in PBS. It demonstrates low cross-reactivity in mammalian cell extracts and E. Coli; each lot is tested for reactivity in immunocytochemistry and western blot (where it detects as little as 5ng protein).

Eukaryotic cell lines

Policy information about cell lines

Cell line source(s)	HeLa-modified (HeLa-M) cells from A. Peden (Cambridge Institute for Medical Research)
Authentication	cells authenticated by STR profiling at the University of Pennsylvania
Mycoplasma contamination	cell lines regularly tested for mycoplasma and confirmed negative
Commonly misidentified lines (See ICLAC register)	no commonly misidentified cell lines were used in the study.
